# House Disinfection

**Published:** 1908-03

**Authors:** Theo. Y. Hull

**Affiliations:** San Antonio, Texas


For the Texas Medical Journal.

### House Disinfection.

BY THEO. Y. HULL, B. S., M. D., SAN ANTONIO, TEXAS.

Having spent the greater part of two years in an endeavor to locate that land—the land of the ideal climate, which many seek and but few find—I have very naturally in my study of tuberculosis been deeply impressed with many things relating thereto that may well call for comment. The question of house disinfection is one that has been most forcibly presented to my mind. In many instances I have been absolutely shocked by the carelessness and ignorance displayed by those who should know better. Tuberculosis will never be stamped out until the general public is taught or compelled to use a few simple sanitary measures. There is such a thing as criminal negligence recognized by the law. Preventive medicine has here one of its widest fields. It is observations of this character that have led me to urge upon the health authorities of the State the imperative necessity of the registration of all tubercular sick.

I may well use as an illustration the history of a single room during a period of much less than one year. In that time it was occupied in succession by four persons suffering with tuberculosis in the last stages. Only one of these used a sputum cup, and this one not until after occupying the room for over two months. Not one of them took any adequate precautions to prevent the dissemination of the germs. All used an open tin pail or can in which to expectorate or a newspaper spread upon the carpet at night for the same purpose. Not a single door or window was screened or any other means taken to prevent the free ingress and egress of flies and mosquitoes. To make matters as bad as possible, and to insure a proper receptacle for the germs, the floor was covered with a very heavy carpet, which was never taken up or cleaned during the time. An ordinary broom, without even the semblance of a damp cloth over it, was used in sweeping. Notwithstanding all this, not anything that could be dignified in any possible way with the name of disinfectant was used. No attempt was made to clean the walls, which had been papered years before, and each occupant in turn was led to believe that no one with consumption had ever occupied the room. At least eight persons, five adults and three children, were with or in attendance upon the four sick ones, and spent a large part of their time in this room. Comment is hardly necessary as to the effect upon the eight well persons. One, indeed, informed me that she trusted in "a power higher than man" to keep her from contracting the disease. Unless immunity has in some fortunate way been acquired, it simply means eight additional cases of tuberculosis with eight new foci for spreading the disease.

The statement "No consumptives taken" greets the traveler in nearly every hotel and boarding house in the South, the West and the Southwest. Notwithstanding this, nearly every one is taken who can pay the price, unless the condition is too self-evident. The floors of the sleeping apartments have almost invariably been covered with heavy carpets, and yet only on the rarest occasions have I seen any suitable sanitary device for sweeping and cleaning.

Very crude ideas exist among the laity as to the liability of individuals to contract the disease. A party with several houses to rent informed me that he would not be afraid to inhale an unlimited quantity of bacilli. I was told by a shrewd business man that a case of tuberculosis had never been known to develop in a native of a certain community. This statement was on a par with that of another business man who insisted that mosquitoes would not develop in the stagnant pools along a certain stream. Persons with such primitive ideas often, as in these instances, stand in the way of progress in sanitary matters. The utter disregard of consequences to the well often shown by the landlords of "furnished cottages" is nothing less than criminal, and should be made so by statutory enactment. Recently a party seeking to rent a cottage was informed by the owner that no "consumptives" had lived in it. To my personal knowledge, the only persons in the house for many months were consumptives, one of whom died in it, another has since died and a third is well on his way to the same end. No means of disinfection have been employed. Another instance will show the extent to which this practice is carried in seemingly respectable quarters. A gentleman leased a furnished house on the assurance of the owner that no sick people had lived in it and that the "invariable carpets" had been recently cleaned. It is needless to repeat what the neighbors said as to the sick occupants, and the carpets on closer examination showed no evidence of ever having been put through any process of cleaning.

These instances of personal knowledge might be multiplied, but such things are of common observation. Some day the public will be rudely awakened to the meaning of criminal negligence. Of all the agencies for the free distribution of the germs of tuberculosis and other diseases, the furnished cottage with its monthly tenant has no equal. Is it any wonder that over 10 per cent of all the people die from the ravages of the so-called white plague or house disease and that post-mortem examinations reveal the rather startling fact that from 60 to 90 per cent of all have had it at some period of their lives.

It is extremely difficult to prevent the spread of diseases when every precaution is taken. A physician whose child was stricken with scarlet fever, on its recovery removed from the house and had the premises, as he supposed, thoroughly fumigated. Yet, a little later a family of several children moved into the place, and every child in due time contracted the fever. If the germs of disease will be disseminated when proper means to prevent it are used, how much greater must be the distribution when no precautions are taken whatever.

In tuberculosis, the average patient is either untrained in taking care of the sputum, or is absolutely indifferent to the welfare of others. Nearly every outside patient I see has been using either a newspaper spread on the carpet or an open can or pail, into which the flies swarm at will, or for the sake of variation the open window or stove hearth serves in place of the sanitary spitting cup. It may seem strange, but in many instances one is compelled to resort to arbitrary measures to prevent the continuance of such unsanitary practices.

Knowing these conditions to exist, as nearly every intelligent person does know them, how readily can we see that some sanitary measures much more stringent and far-reaching than any we have on statute books or in town ordinances are imperative.

With this survey of the field in which work is so urgently needed, let us turn to some of the means employed by the laity in an attempt at sanitation. The first thing one notices is the extremely primitive methods followed. A box of matches, a handful of sulphur, a sulphur candle, a weak solution of carbolic acid and an ounce or two of formaldehyde sprinkled over a dry floor will disinfect, we are told, a room of any dimensions with little attention paid to closing apertures. A lady and her children spent the summer on a ranch. She was given a room that was said to be thoroughly disinfected. A party having tuberculosis in the second stage had occupied the room for several months. A box of matches was burned in the room to render it thoroughly germ free.

In another instance a party rented a large furnished house with the intention of taking weekly roomers. Every room in the building within ten months had been occupied by consumptives. I asked him if he had considered the need of fumigation. He intended burning a little sulphur in each room. He may have done so, but the windows were never closed, and the sign "furnished rooms" was hung out the next morning. Supposedly healthy people have occupied the rooms ever since—men, women and children.

In a Southern city where the city fathers passed an ordinance requiring all houses to be fumigated immediately on being vacated by persons sick of tuberculosis, but failed to prescribe any method of procedure or to designate any officer to attend to such matters. I examined a house that the owner declared had been thoroughly disinfected the previous day. I could detect no odor of disinfectants and saw no evidence of it save a few spots on the floor, where evidently a little formaldehyde had evaporated. Every family who had lived in this house had one or more persons sick with tuberculosis.

The question of checking the spread of tuberculosis is a most serious one, and one that calls for the application of the highest intelligence, but what can we expect to result if such primitive methods of fumigation are not forbidden and the common practice of allowing well people to occupy apartments just vacated by the sick is not checked by law. Our statute books are filled with laws designed to prevent one man from despoiling his neighbors, either his property or his good name. Does not he who for gain or for want of care places another in such a position that he contracts a fatal disease despoil him of something more valuable than his property, and yet he can do this with impunity, for there is no law to reach him. If such practices as I have described existed in only a few localities, it would not be so serious a matter, but, unfortunately, they are all but universal in this country.

In many cities measures have been taken for several years to check the spread of tuberculosis, but few of them are effective, and none of them place any degree of responsibility upon the owner, agent or lessee that will make them so. In the city of Washington (to 1906) the health department, on receipt of a death certificate showing tuberculosis as the cause of death, offered to disinfect free of charge the room and bedding occupied and used by the deceased, but this was not compulsory, and no attention was given to rooms occupied by consumptives and vacated by them during life. Of course, no regulation embodying this latter point can be made effective until we have a systematic registration of every person suffering from tuberculosis and his whereabouts made a matter of record. In Asheville a town ordinance makes it compulsory to fumigate rooms occupied by consumptives when vacated, but renders it of very little practical value by its failure to prescribe a definite method or methods of disinfection, and it further failed in not designating some official to carry it into effect. When these matters are left to the public or the property owner, the least expensive method and the one most easily carried out will be used. The question of effectiveness will not receive any great consideration.

That this failure to effectively disinfect premises occupied by the tubercular sick, or to disinfect them at all, is a serious evil all must admit, and an equal evil in any other walk of life would be considered as criminal and penalties provided for its punishment. There is but one way to correct the evil, and that is by enactment of rigid State regulations. I may not be my brother's keeper, but I must not be his destroyer.

As to the question of legislation, any law regulation or ordinance to be effective must embody the following points:

1. It should require under sufficient penalty all rooms occupied by persons suffering from tuberculosis to be fumigated thoroughly immediately on being vacated. In the case of furnished rooms, it should require the bedding, carpets, rugs and everything used by the patient to be treated in the same way. It will not be sufficient to advise this to be done. It must be made obligatory upon the owner, agent or lessee, and proper punishment prescribed in case of failure to do so. It has been my experience, and I believe the experience of most physicians, that many people will not use disinfectants at all unless compelled by law. The owner of a row of tenant houses as well as the owner of a few furnished rooms will count the cost and trouble, and will hold this of far greater weight than the danger to the other fellow.

2. In order to render such an ordinance effective it must prescribe a definite method or methods of disinfection. To leave this to the judgment or avarice of the landlord or agent means to render it non-effective, as I have shown above. My own preference is for formaldehyde poured over permanganate of potash in the presence of sufficient moisture in the room, after the manner prescribed by the *Journal A. M. A.*, July 14, 1906, page 139, and tested by the State Boards of Health of Maine, Illinois and New Hampshire. This requires for every 1000 cubic feet of air space one pint of a 40 per cent solution of formaldehyde and four to six ounces of permanganate of potash. A solution of mercuric bichloride 1 to 1000 should be used to disinfect the woodwork, floors and furniture. But, whatever method is prescribed, a sufficient quantity of the disinfectant should be used to insure effective work.

3. To make disinfection obligatory upon the proper persons and to prescribe the method of doing it will not always accomplish the result. To render the ordinance doubly effective, it must go further and designate some official as a "public fumigator," whose duty it should be to have the work carried out under his direction and to be personally responsible for its thoroughness. The cost should be assessed against the landlord or tenant, as the case may be. This is essentially right and proper. It is not to be supposed that any one wishes another to contract a disease from which he is dying, and the assurance that such will not be the case should be rendered doubly sure.

4. No owner or agent should be permitted to rent any premises, either cottage or room, empty or furnished, that have been occupied by consumptives in any stage of the disease without first providing a certificate signed by the proper official showing when and how said premises were disinfected. This should be a matter of record. The extent to which persons afflicted with tuberculosis flock to certain cities and parts of the country renders some such regulation absolutely imperative.

5. To enable the authorities to make the other sections of a city ordinance more effective, there should be a complete registration of each person suffering from tuberculosis, and his or her whereabouts should be a matter of record from month to month. My experience with owners of "houses to let" leads me to urge and insist upon some form of record to which the respective lessee may appeal. It is not only desirable but an urgent necessity in the fight against tuberculosis.

6. Another section I would add in the way of a preventive measure. We have ordinances against expectorating upon the public sidewalk, in a public hall or in any public conveyance. It may not be going too far to urge some form of sanitary "spitting cup" for the use of each individual in his home. The large number of untrained and unthinking sick make some such regulation necessary.

In conclusion let me add, many object to this form of regulation as restricting to too great a degree the rights and privileges of the individual. The same may be said of the crusade in all the great cities to secure a better milk supply. The same has been said and is being reiterated in the struggle to secure pure food and drug legislation. No great reform has ever been accomplished without criticism and opposition. Neither will this, the greatest of works of preventive medicine, the stamping out of tuberculosis, be accomplished without a struggle. It may cause hardships to some, but in certain emergencies they must be borne for the public good, and no greater good can come to a people. One hundred and fifty thousand deaths a year from one cause amounts to a national scourge, and its concomitant evils constitute a national calamity. "To promote the general welfare," the preamble to our national Constitution, declares to be one of the objects of the government. What can promote the general welfare to so great a degree as to save one million people of this land annually from the evils of that most loathsome of all diseases—tuberculosis?

